# Tetraspanin CD9 Limits Mucosal Healing in Experimental Colitis

**DOI:** 10.3389/fimmu.2017.01854

**Published:** 2017-12-19

**Authors:** María Laura Saiz, Danay Cibrian, Marta Ramírez-Huesca, Daniel Torralba, Olga Moreno-Gonzalo, Francisco Sánchez-Madrid

**Affiliations:** ^1^Immunology Service, Hospital de la Princesa, Universidad Autónoma de Madrid, Instituto de Investigación Sanitaria del Hospital Universitario de La Princesa, Madrid, Spain; ^2^Department of Vascular Biology and Inflammation, Centro Nacional de Investigaciones Cardiovasculares (CNIC), Madrid, Spain; ^3^CIBER Cardiovascular, Madrid, Spain

**Keywords:** tetraspanins, CD9, mucosal healing, dextran sodium sulfate, colitis

## Abstract

Tetraspanins are a family of proteins with four transmembrane domains that associate between themselves and cluster with other partner proteins, conforming a distinct class of membrane domains, the tetraspanin-enriched microdomains (TEMs). These TEMs constitute macromolecular signaling platforms that regulate key processes in several cellular settings controlling signaling thresholds and avidity of receptors. In this study, we investigated the role of CD9, a tetraspanin that regulates major biological processes such as cell migration and immunological responses, in two mouse models of colitis that have been used to study the pathogenesis of inflammatory bowel disease (IBD). Previous *in vitro* studies revealed an important role in the interaction of leukocytes with inflamed endothelium, but *in vivo* evidence of the involvement of CD9 in inflammatory diseases is scarce. Here, we studied the role of CD9 in the pathogenesis of colitis *in vivo*. Colitis was induced by administration of dextran sodium sulfate (DSS), a chemical colitogen that causes epithelial disruption and intestinal inflammation. CD9^−/−^ mice showed less severe colitis than wild-type counterparts upon exposure to DSS (2% solution) and enhanced survival in response to a lethal DSS dose (4%). Decreased neutrophil and macrophage cell infiltration was observed in colonic tissue from CD9^−/−^ animals, in accordance with their lower serum levels of TNF-α, IL-6, and other proinflammatory cytokines in the colon. The specific role of CD9 in IBD was further dissected by transfer of CD4^+^ CD45RB^hi^ naive T cells into the Rag1^−/−^ mouse colitis model. However, no significant differences were observed in these settings between both groups, ruling out a role for CD9 in IBD in the lymphoid compartment. Experiments with bone marrow chimeras revealed that CD9 in the non-hematopoietic compartment is involved in colon injury and limits the proliferation of epithelial cells. Our data indicate that CD9 in non-hematopoietic cells plays an important role in colitis by limiting epithelial cell proliferation. Future strategies to repress CD9 expression may be of therapeutic benefit in the treatment of IBD.

## Introduction

Inflammatory bowel disease (IBD) defines a group of intestinal disorders, principally, ulcerative colitis (UC) and Crohn’s disease (CD). Both diseases are characterized by chronic inflammation of the gastrointestinal tract interspersed with relapsing phases (1). Much progress has been made in understanding UC and CD disease mechanisms, for example, through genome-wide association studies in patients; however, these diseases remain incompletely understood. Identified genetic risk loci have revealed defects in IBD patients affecting genes crucial for intestinal homeostasis, including epithelial barrier function, restitution, and wounding (2). Moreover, recent clinical studies have revealed mucosal healing (MH) as the major prognostic predictor of long-term remission in IBD patients (3, 4), suggesting that epithelial regeneration is critical to improving IBD therapy (5).

Tetraspanins are proteins that span the cell membrane four times and play an important role in plasma membrane organization through the formation of tetraspanin-enriched microdomains, which enable them to associate with multiple proteins, including other tetraspanins (6). The tetraspanin CD9 is broadly expressed on the surface of several cell types, including many malignant tumor cells, as well as normal hematopoietic, endothelial, and epithelial cells (7, 8). Soon after its identification, CD9 was found to associate with several integrins (9), enabling CD9 to exert pro- or anti-migratory effects (10). CD9 can also interact with the immunoglobulin superfamily members EWI-2 and EWI-F (11), DDR1 (12), other tetraspanins (e.g., CD81 and CD151) (13), claudin-1 (14), ADAM10 (15), and ADAM17 (16) metalloproteases, epidermal growth factor receptor (EGFR) (17), and membrane-bound EGFR ligands (18, 19). Moreover, CD9 has been reported to regulate endothelial nanoscopic organization and expression levels of ICAM-1 and VCAM-1 upon TNF-α activation, enabling formation of the docking structure required for leukocyte extravasation (20, 21). Anti-CD9 agonistic antibodies or ectopic expression of CD9 both exert an antiproliferative effect on human colon carcinoma cell lines (22). However, the role of CD9 in IBD has not been previously addressed *in vivo*. Here, we show that CD9 acts as a limiting factor for epithelial regeneration and colonic MH in dextran sodium sulfate (DSS)-induced colitis.

## Materials and Methods

### Mice

Experiments were performed with sex and age matched (8- to 12-week old) CD9^−/−^ and WT mice on the C57BL/6 background. CD9^−/−^ mice have been described previously (23). Rag1^−/−^ mice (24) used in the adoptive transfer colitis model were kindly provided by Dr. J. M. Fernández-Granado (CNIC). For chimeric reconstitution experiments, B6SJL CD45.1 mice (Jackson Laboratories) were used. All animals were housed in pathogen-free conditions at the CNIC animal facility. Experimental procedures were approved by the local research ethics committee and conformed to EU Directive 2010/63EU and Recommendation 2007/526/EC, enforced in Spanish law under Real Decreto 53/2013.

### Induction and Assessment of DSS-Induced Colitis

Dextran sulfate sodium salt (DSS, MP Biomedicals; *M*_W_ = 36,000–50,000) was dissolved at 2 or 4% (w/v) in sterile drinking water provided to mice *ad libitum*. Mice were checked daily for development of colitis by monitoring body weight, fecal occult blood (Hemoccult II Sensa; Beckman Coulter) or gross rectal bleeding, and stool consistency. Overall disease severity was assessed by a clinical scoring system defined as follows: weight loss: 0 (no loss), 1 (1–5%), 2 (5–10%), 3 (10–20%), and 4 (>20%); stool consistency: 0 (normal), 2 (loose stool), and 4 (diarrhea); and bleeding: 0 (no blood), 1 (Hemoccult positive), 2 (Hemoccult positive and visual pellet bleeding), and 4 (gross bleeding, blood around anus). At the end of the experiment, tissues were fixed in 10% neutral buffered formalin (Bio Optica) for 24 h and transferred to 70% ethanol. After embedding in paraffin, transverse sections (4–5 µm) of proximal and distal colon were stained with H&E for histological studies. Images were digitized using Hamamatsu Nanozoomer 2.0 RS scan and NDP.scan 2.5 digitization software. Three images of two serial sections cut at a separation of 100 µm (six sections in total) were evaluated for each mouse for each part of the colon (proximal and distal). Histological scoring evaluated inflammation severity, crypt damage, and ulceration. Inflammation severity was scored as follows: 0, rare inflammatory cells in the lamina propria; 1, increased numbers of granulocytes in the lamina propria; 2, confluence of inflammatory cells extending into the submucosa; 3, transmural extension of the inflammatory infiltrate. Crypt damage was scored as follows: 0, intact crypts; 1, loss of the basal one-third; 2, loss of the basal two-thirds; 3, entire crypt loss; 4, change of epithelial surface with erosion; 5, confluent erosion. Ulceration was scored as follows: 0, absence of ulcers; 1, 1–2 ulceration foci; 2, 3–4 ulceration foci; 3, confluent or extensive ulceration. Scores for each parameter were summed to give a maximum histological score of 11.

### T Cell-Mediated Colitis

Naive CD4^+^ T cells were sorted (FACSaria sorter, BD) from single-cell spleen suspensions of CD9^−/−^ or WT mice. Live cells were isolated after labeling with antibodies to CD4, CD62, CD25, and CD45RB (eBiosciences) and hoescht 33258. Cells were transferred to recipient mice (4–5 × 10^5^ cells per mouse) by intraperitoneal injection.

### Bone Marrow Chimeras

Bone marrow transfer was used to create chimeric mice in which genetic deficiency for CD9 was confined to either circulating cells (CD9^−/−^ > WT) or nonhematopoietic tissue (WT > CD9^−/−^). Briefly, bone marrows were collected from femur and tibia of congenic WT donor mice (expressing CD45.1 leukocyte antigen) or CD9^−/−^ and WT donor mice (expressing CD45.2 leukocyte antigen) by flushing with PBS. Erythrocytes were lysed (ACK lysis buffer, Lonza) for 1 min on ice. After a washing step, cells were resuspended in PBS at 1 × 10^8^/ml. This cell suspension (100 µl) was injected intravenously into 13 Gy-irradiated recipient mice 48 h postirradiation. Four chimera groups were generated: WT > WT (WT cells expressing CD45.1 into WT mice expressing CD45.2); WT > CD9^−/−^ (WT cells expressing CD45.1 into CD9^−/−^ mice expressing CD45.2); WT > WT (WT cells expressing CD45.2 into WT mice expressing CD45.1); CD9^−/−^ > WT (CD9^−/−^ cells expressing CD45.2 into WT mice expressing CD45.1). Bone marrow reconstitution was verified after 8 weeks by staining for CD45.1 or CD45.2 in blood cells with anti-CD45.1 or anti-CD45.2 specific antibodies (BD Biosciences).

### *In Vivo* Permeability Assay

Food was withdrawn overnight and mice were gavaged with the permeability tracer FITC-dextran (MW 4,000; Sigma-Aldrich) at 60 mg/100 g body weight. After 4 h, blood was collected by heart puncture and serum FITC-dextran was measured with a fluorescence spectrophotometer (Fluoroskan Ascent; Thermo Labsystems) using emission and excitation wavelengths of 490 and 520 nm, respectively. FITC-dextran concentration was determined from a standard curve generated by serialdilution.

### Isolation and Flow Cytometry Analysis of Colonic Leukocytes

Colons were dissected longitudinally, washed several times with PBS to remove feces, and cut into small pieces. Samples were digested with 0.25 mg/ml Liberase TM (Roche), 50 µg/ml DNAseI (Roche), and 1 mM DTT diluted in Hank’s Balanced Solution for 30 min at 37°C. At the end of the incubation period, enzyme activity was blocked by adding 50 ml PBS supplemented with 0.5% BSA and 0.05 mM EDTA (PBS–BSA–EDTA), and the sample was mechanically disrupted by passing through a 70-μm cell strainer to obtain a cell suspension. When only epithelial cells were required, samples were incubated in 5 mM EDTA, 1 mM DTT for 20 min before enzyme digestion. Before all staining procedures, colon cell suspensions were incubated with anti-mouse FcRII/III (clone 2.4G2, TONBO Biosciences) for 10 min at 4°C in PBS-BSA-EDTA. Flow cytometry analysis of DSS-induced inflammation was performed with anti-mouse antibodies to the following antigens: CD45 (BD Horizon) and CD11b, CD64, and Ly6G (BD Pharmingen). For epithelial cell proliferation analysis, antibodies were used targeting EpCAM (Biolegend) and Ki67 (BD Pharmingen). Absolute cell numbers were obtained using TruCount Tubes (BD Biosciences). Cell samples were acquired in a FACSCanto Flow Cytometer (BD Biosciences), and the data were analyzed with FlowJo (Tree Star) or FACSDiva (BD Biosciences) software.

### Flow Cytometric Bead Array (CBA)

Serum TNF-α, IL-6, and IFNγ were determined using the mouse Th1/Th2/Th17 BD CBA.

### RNA Extraction and Real-time Quantitative PCR

RNA was isolated by disrupting colon tissue samples with TRIzol Reagent (1 ml per 50–100 mg tissue, Qiagen) and homogenizing in a tissue disruptor (Ika ultra-turrax T10 homogenizer). DSS traces were removed by the LiCl method (Ambion). Residual DNA contamination was eliminated with the Turbo DNA-free Kit (Ambion). Total RNA (1 µg) was reverse transcribed to cDNA with a Reverse Trancription Kit (Applied Biosystems). Quantitative PCR was then performed in an AB7900_384 (Applied Biosystems) using SYBR Green (Applied Biosystems) as the reporter. Gene-specific primers used are listed in Table S1 in Supplementary Material. Expression of each gene of interest was normalized to housekeeping gene GAPDH. Data are presented as relative fold differences calculated by the 2^−ΔΔCt^ method.

### *In Vitro* T Cell Differentiation

Naive CD4^+^ T cells were obtained by incubating single-cell suspensions of spleen and lymph nodes with biotinylated antibodies to CD8, CD16, CD19, F4/80, Gr-1, MHC class II (I-Ab), CD11b, CD11c, and DX5 followed by incubation with Streptavidin Microbeads (MACS, Miltenyi Biotec). CD4^+^ T cells were isolated by negative selection in an auto-MACSTM Pro Separator (Miltenyi Biotec). Next, cells were activated with plate-bound anti-CD3 (5 µg/ml) and anti-CD28 (2 µg/ml) in RPMI 1640 medium (Sigma-Aldrich) supplemented with 10% FCS, 2 × 10^−3^ M l-glutamine, 100 U/ml penicillin, 100 µg/ml streptomycin, 50 µM 2-mercaptoethanol, and the corresponding cytokine cocktail: for Th0, anti-IFNγ (4 µg/ml), anti-IL-4 (4 µg/ml), and IL-2 (10 ng/ml); for Th1 anti-IL-4 (4 µg/ml), IL-12 (10 ng/ml), and IL-2 (10 ng/ml); for Th17 anti-IFNγ (4 µg/ml), anti-IL-4 (4 µg/ml), IL-6 (20 ng/ml), IL-23 (10 ng/ml), and TGF-β1 (5 ng/ml); and for Treg anti-IFNγ (4 µg/ml), anti-IL-4 (4 µg/ml), and TGF-β1 (10 ng/ml). After 72 h of culture, IFNγ, IL-17, or IL-10 in the supernatant were measured by ELISA (Ready-SET-Go, eBiosciences). For FACS analysis, intracellular cytokine staining was preceded by restimulation for 4 h with 50 ng/ml phorbol dibutyrate (PMA) and 500 ng/ml ionomycin in the presence of brefeldin A (1 µg/ml) (BD Biosciences).

### Immunofluorescence and Immunohistochemical Analysis

For IF and IHC staining, colon sections were deparaffinized, boiled in antigen retrieval solution (10 mM Tris Base, 1 mM EDTA Solution, 0.05% Tween 20, pH 9.0 for Ki67 and 10 mM sodium citrate, 0.05% Tween 20, pH = 6 for caspase-3), and incubated with the rabbit monoclonal anti-mouse Ki67 primary antibody (Master Diagnostica, clon SP6) for IF or anti active caspase-3 rabbit polyclonal antibody for IHC (R&D system, catalog AF835). Bound antibodies were detected with a goat anti-rabbit 647 (ThermoFischer Scientific) for IF or the anti-rabbit EnVision FLEX-HRP detection system (Agilent) for IHC. Staining was developed with DAB substrate (Dako K3468), and slides were counterstained with Mayers Hematoxylin. Ki67 and active caspase-3 staining in epithelial cells were quantified in the whole colon sections from each DSS-treated mouse (4–5 mice per group). Image J (1.46r) was used to measure Ki67 positive individual nuclei and to measure caspase-3 intensity relative to the total area corresponding to the complete epithelial layer in each image.

### Statistical Analysis

Data are presented as mean ± SD. Normal data distribution was assessed with the Kolmogorov Smirnov test, and the statistical significance of between-group differences was assessed by one-tailed unpaired Student’s *t*-test or one-way ANOVA with Newman–Keuls multiple comparison *t*-test, as required. All statistical analyses were performed with GraphPad Prism (GraphPad Software Inc.).

## Results

### CD9^−/−^ Mice Are Protected against DSS-Induced Colonic Injury

**Figure 1:**
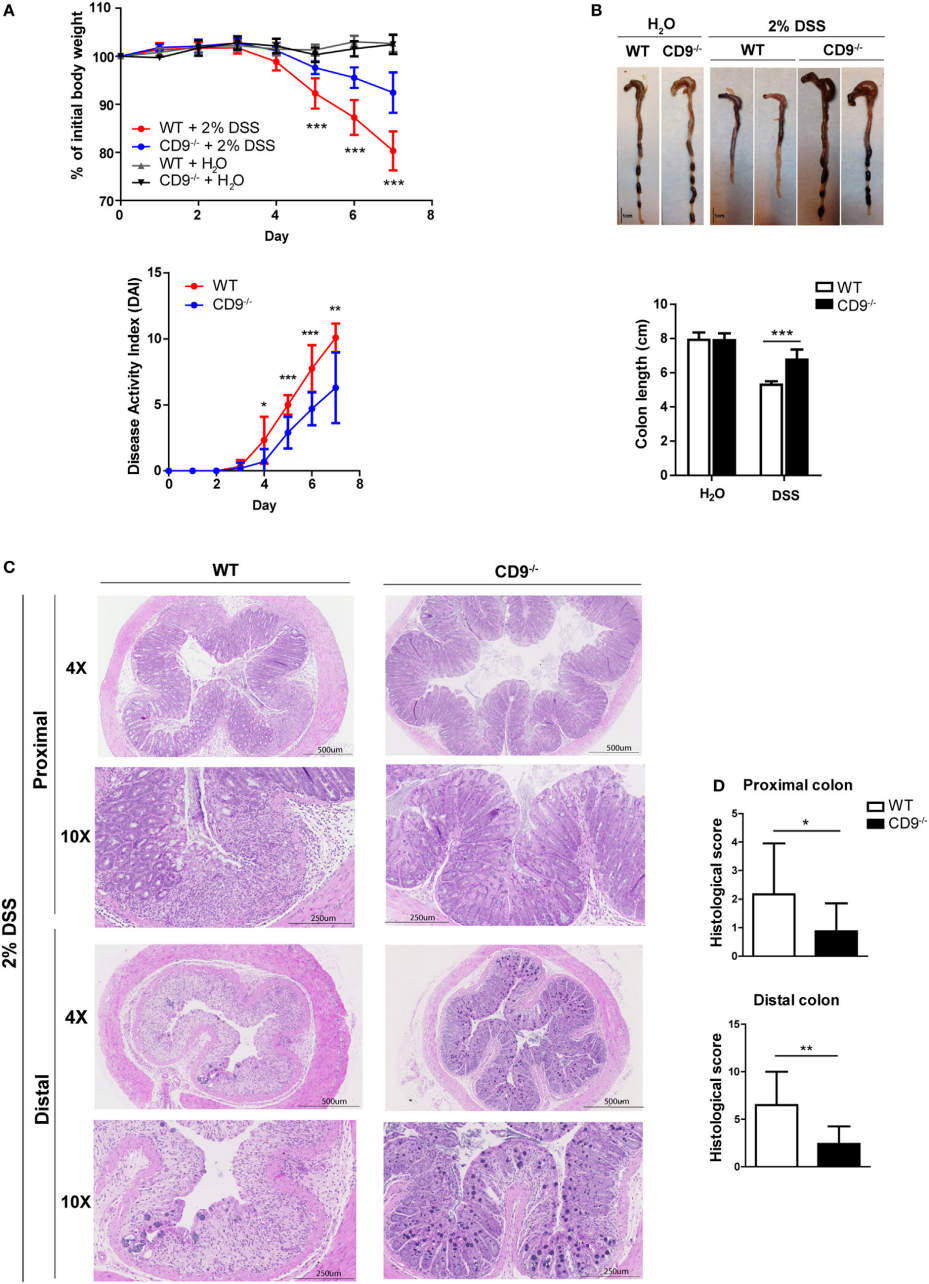
CD9-deficiency reduces sensitivity to dextran sodium sulfate (DSS)-induced colitis. **(A)** *Top*, body-weight loss in WT and CD9^−/−^ mice after administration of 2% DSS in drinking water for 7 days. Controls for each genotype were administered with unadulterated drinking water. *Bottom*, disease activity index (DAI) score in WT and CD9^−/−^ mice after administration of 2% DSS for 7 days. *n* = 10–12 per group; *P < 0.05; **P < 0.005; ***P < 0.001, unpaired *t*-test. **(B)** Macroscopic colon damage in DSS-treated WT and CD9^−/−^ mice. *Top*, Colon shrinkage. *Bottom*, changes in colon length. Representative colons are shown of *n* = 10–12 mice per group. **(C)** Representative photomicrographs of proximal colon (near the cecum) and distal colon (near the anus) from WT and CD9^−/−^ mice at day 7 of DSS administration (H&E; magnifications: 4× and 10×). **(D)** Histological scores obtained from H&E-stained proximal and distal colon tissue sections from DSS-treated WT and CD9^−/−^ mice. Data are pooled from two independent experiments (*n* = 4). Values represent mean ± SD of the mean: *P < 0.05; **P < 0.005; ***P < 0.001, unpaired *t*-test.

To explore the function of CD9 in colitis development, we challenged CD9^−/−^ and WT mice with the toxic compound DSS (2% solution) in drinking water for 7 days. CD9^−/−^ animals lost less than 10% of their initial body weight, whereas WT counterparts lost around 20% (Figure 1A, top). To monitor disease activity, we recorded a daily disease activity index (DAI) combining weight loss, stool consistency, and bleeding. From day 4, DAI values were lower in CD9^−/−^ mice than in WT counterparts (Figure 1A, bottom). Autopsy revealed that DSS-treated CD9^−/−^ mice had significantly larger colons than WT counterparts (Figure 1B). Histology revealed a better preservation of tissue architecture in CD9^−/−^ mice, in both the proximal and the distal colon. DSS-treated WT animals showed more pronounced epithelial denudation, crypt distortion, leukocyte infiltration of the lamina propria, and submucosal swelling (Figure 1C). Histological sections were scored for the severity of DSS-induced inflammation as described in Section “Materials and Methods.” In both proximal and distal colon, histological scores were lower in CD9^−/−^ mice than in WT mice, with the difference more pronounced in the distal colon (Figure 1D).

### CD9 Exacerbates Tissue Injury and Decreases Mouse Survival after a Lethal DSS Dose

**Figure 2:**
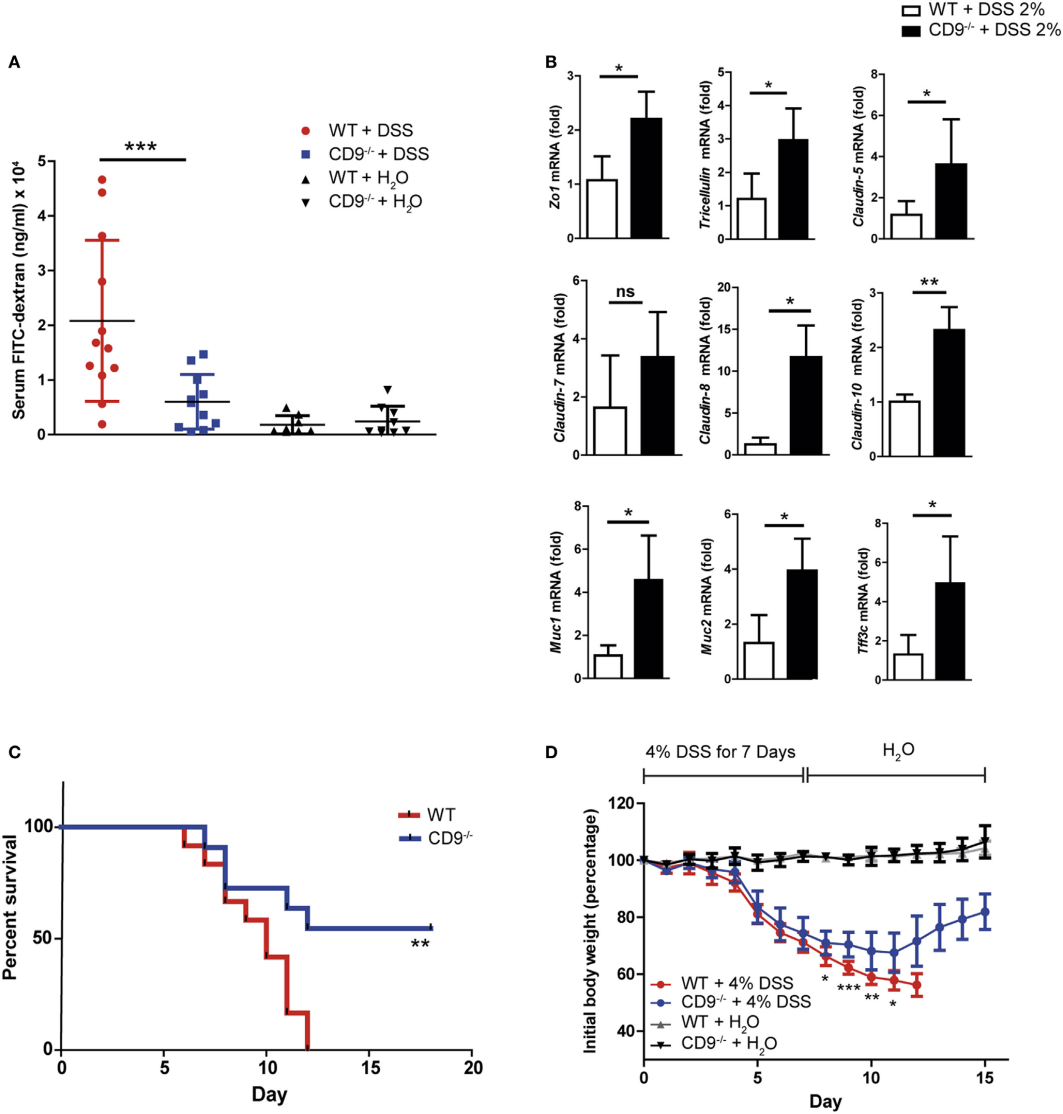
Enhanced epithelial barrier integrity and survival in CD9^−/−^ mice after dextran sodium sulfate (DSS) challenge **(A)** *In vivo* colon permeability, indexed from the serum level of 4 kDa FITC-dextran 4 h after feeding by gavage. Data are pooled from two independent experiments, *n* = 5–6 animals per group. Data were analyzed by one-way ANOVA and the Newman–Keuls multiple comparison test; ***P < 0.001. **(B)** qPCR analysis of tight junction and mucin gene expression in colon samples after 7 days of DSS exposure. Data are from one experiment repeated two times with similar results. **(C)** Kaplan–Meier survival for WT and CD9^−/−^ mice given 4% DSS in drinking water. **P < 0.01, Log-rank (Mantel–Cox) test. **(D)** Percentage of initial body weight of WT and CD9^−/−^ mice after 7-day intake of 4% DSS chased by unadulterated water. *n* = 11–12 per group: *P < 0.05; **P < 0.005; ***P < 0.001; unpaired *t*-test for WT and CD9^−/−^ groups in **(B,C)**.

Intestinal epithelial integrity is necessary for efficient defense against intraluminal toxins, antigens, and enteric bacteria. Cells are tightly joined in a healthy epithelium, and transepithelial permeability can thus be determined as an index of epithelial integrity. To monitor gut barrier function *in vivo*, we treated CD9^−/−^ and WT animals with 2% DSS for 7 days and then orally administered 4KDa FITC-Dextran. Fluorescence spectrophotometry detection of serum FITC after 4 h revealed markedly lower gastrointestinal permeability in CD9^−/−^ mice than in WT mice (Figure 2A). Serum FITC levels in non-treated animals showed no significant between-genotype differences and remained below 5 μg/ml, consistent with an intact intestinal barrier function in the steady state (Figure 2A). Consistent with the FITC-Dextran data, qPCR of colon samples from DSS-treated CD9^−/−^ mice revealed elevated expression of genes encoding epithelial tight junction proteins, such as ZO-1, tricellulin, and claudin family members (Figure 2B). CD9^−/−^ colon also showed elevated expression of genes encoding epithelial globet cell proteins, such as the secretory mucin glycoproteins MUC1, MUC2, and trefoil factor 3, indicating normal intestinal function (Figure 2B). In a further approach, we exposed mice to a lethal DSS dose (4%) for 7 days followed by unadulterated drinking water for a further 8 days. At the end of the experiment all WT mice had died, whereas only 45% of the CD9^−/−^ group were dead (Figure 2C). Moreover, the surviving CD9^−/−^ mice showed a recovery in body weight (Figure 2D). These results show that CD9 impedes epithelial repair and contributes to colon injury at both sublethal and lethal DSS doses.

### Reduced Myeloid Cell Infiltration and Proinflammatory Cytokine Expression in the Colon of CD9^−/−^ Mice

**Figure 3:**
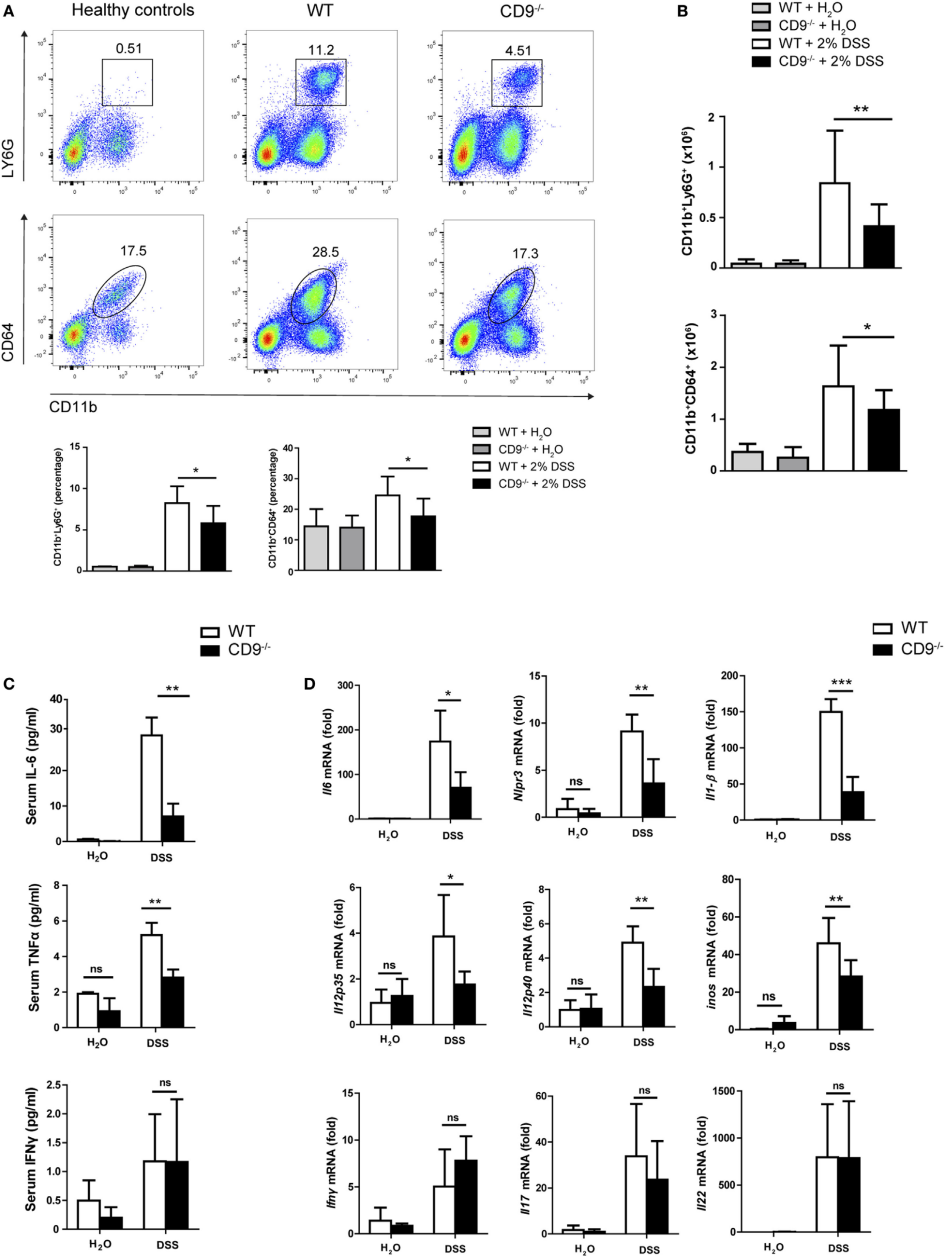
CD9^−/−^ mice exhibit lower leukocyte infiltration and proinflammatory cytokines in serum and colon after 2% dextran sodium sulfate (DSS) administration. **(A)** Flow cytometry analysis of whole colon from WT and CD9^−/−^ mice after 7-day 2% DSS intake. Representative dot plots and percentage quantification of CD45^+^ populations show similar DSS-induced infiltration by neutrophils (Ly6G^+^) and macrophages (CD64^+^) in both genotypes. **(B)** Total neutrophil and macrophage numbers in the CD45^+^-gated population, determined by TruCount Tubes. Data are pooled from two independent experiments. For **(A,B)**, *n* = 6–7 mice per group with two repeats and analysis by one-way ANOVA and the Newman–Keuls multiple comparison test. *P < 0.05; **P < 0.005; ***P < 0.001. **(C)** Serum levels of TNF-α, IL-6, and IFNγ measured by cytometric bead array assay. **(D)** qPCR analysis of colonic proinflammatory cytokine mRNA expression. Bars denote the mean ± SD of *n* = 4–5 mice per genotype. Data from **(C,D)** were analyzed by two-way ANOVA and the Bonferroni multiple comparison test.

To characterize the immune mechanisms of colonic mucosa damage, we analyzed CD9^−/−^ and WT colon cells by flow cytometry. After 7-day exposure to 2% DSS, CD9^−/−^ colon showed markedly lower neutrophil and macrophage infiltration than WT colon (Figures 3A,B). In contrast, in non-treated mice, gut populations of these immune cell subsets were comparable between genotypes (Figures 3A,B), as were mesenteric lymph nodes, intraepithelial lymphocyte and lamina propria populations (Figure S1 in Supplementary Material). DSS-treated CD9^−/−^ mice also had lower serum levels of IL-6 and TNFα than WT mice, whereas IFNγ was similarly increased in response to DSS in both genotypes (Figure 3C). Analysis of colon samples by qPCR revealed lower DSS-induced levels of IL-6, IL-1β, NLPR3, iNOS, IL-12p35, and IL-12p40 mRNA in CD9^−/−^animals, whereas IL-17, IL-22, and IFNγ showed no significant between-genotype differences (Figure 3D).

### CD9 Expressed on CD4^+^ T Cells Does Not Contribute to Immune-Cell Adoptive Transfer-Mediated Colitis

**Figure 4:**
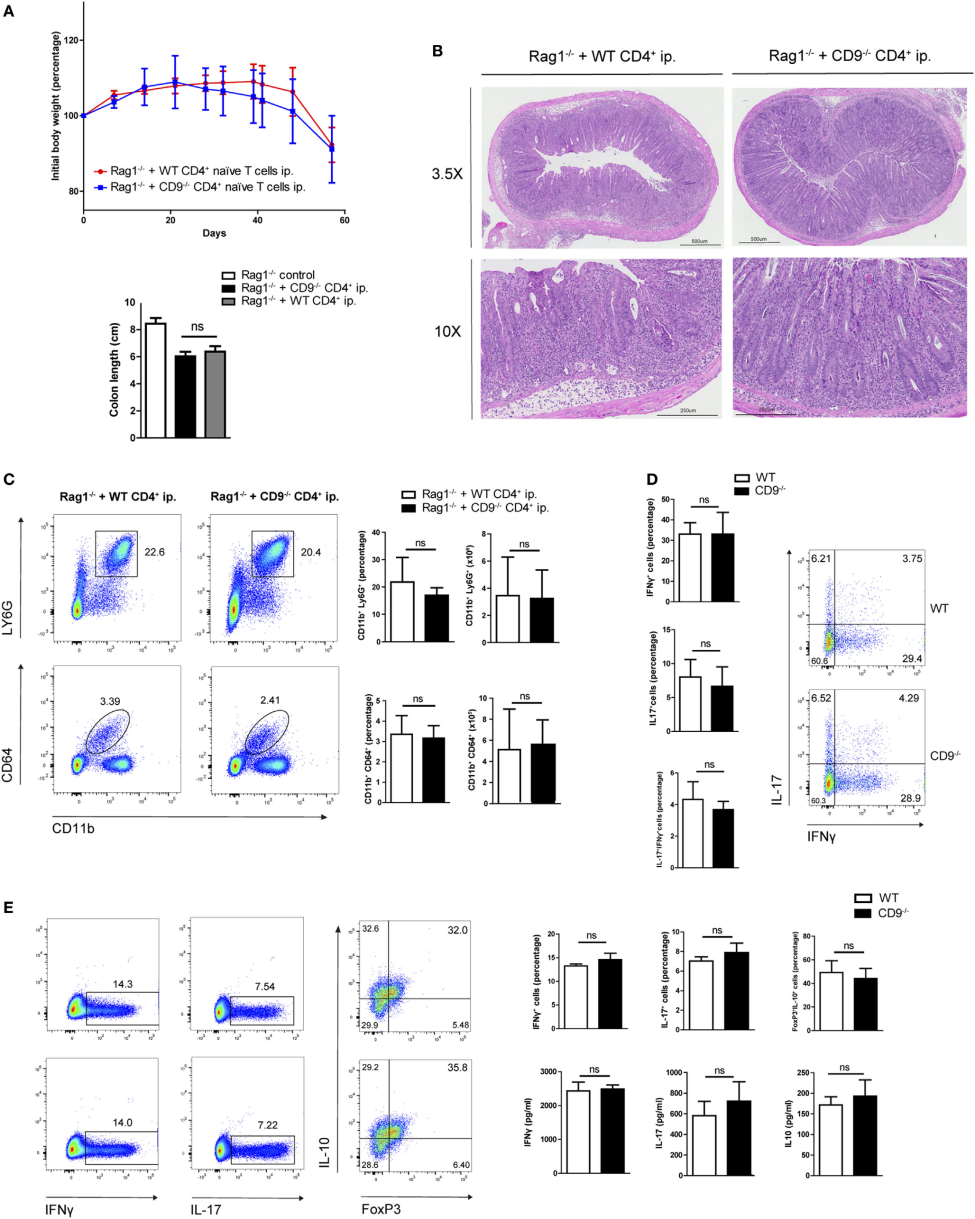
CD4^+^ T cell-expressed CD9 does not contribute to adoptive transfer-mediated colitis or T cell differential subset skewing. **(A)** *Top*, body weight after intraperitoneal adoptive transfer of CD4^+^CD45RB^hi^ CD62L^+^CD25^−^ T cells from WT and CD9^−/−^ donors into Rag1^−/−^ recipients. *Bottom*, colon length at sacrifice on day 57. Data are from a representative experiment repeated three times with similar results. *n* = 5–6 mice per group; unpaired *t*-test. ns, non significant. **(B)** Representative 3.5× and 10× magnification H&E-stained colonic sections from Rag1^−/−^ mice injected with WT and CD9^−/−^ CD4^+^ cells, showing transmural infiltration affecting all colon layers in both settings. **(C)** FACS analysis of myeloid cell infiltration. Representative dot plots are shown on the left; quantification of CD45^+^-gated cell percentages and total numbers is shown on the right. **(D)** Flow cytometry analysis of intracellular staining for IFNγ and IL-17 in T cells from the mesenteric lymph nodes (MLNs) of RAG1^−/−^ mice 2 months after CD4^+^ T cell transfer. Cells were cultured for 72 h on an anti-CD3/CD28-coated plate and brefeldin A was added for the last 4 h. Representative dot plots and bar quantifications are shown of CD4^+^CD25^+^-gated cells. *n* = 5–6 mice per group; unpaired *t*-test. **(E)** *In vitro* T cell differentiation toward Th1, Th17, and Treg CD4^+^ T cell subsets. Representative dots plots are shown of intracellular IFNγ, IL-17, and IL-10 in sorted populations, with quantification on the right (top row). Cytokine release was quantified by ELISA (bottom). Data are from a representative independent experiment of three performed and are presented as mean ± SD. *n* = 5 per genotype; unpaired *t*-test.

To further explore possible CD9-mediated immune mechanisms in IBD, we used an alternative model of colitis induced by intraperitoneal transfer into Rag1^−/−^ mice of CD4^+^CD62L^+^CD25^−^CD45RB^hi^ naive T cells sorted from WT or CD9^−/−^ mice. Body weight was recorded over 2 months, showing no between-group differences (Figure 4A, top). Colon shortening was also similar in both genotypes (Figure 4A, bottom). Consistent with these findings, histological analysis revealed a similar extent of transmural inflammation in injected animals (Figure 4B), and flow cytometry showed similar increases in neutrophil and macrophage infiltration (Figure 4C). Restimulation of mesenteric lymph node CD4^+^ cells with CD3/CD28 revealed no significant differences in Th1 and Th17 effector cell populations or cytokine production (Figure 4D). Likewise, no between-genotype differences were observed in the percentages of Th1, Th17, and Treg cells upon *in vitro* polyclonal differentiation of CD4^+^ naive T cells from CD9^−/−^ and WT mice (Figure 4E).

### CD9^−/−^ Bone Marrow Cells Transplanted into WT Mice Do Not Provide Protection against Colonic Injury

**Figure 5:**
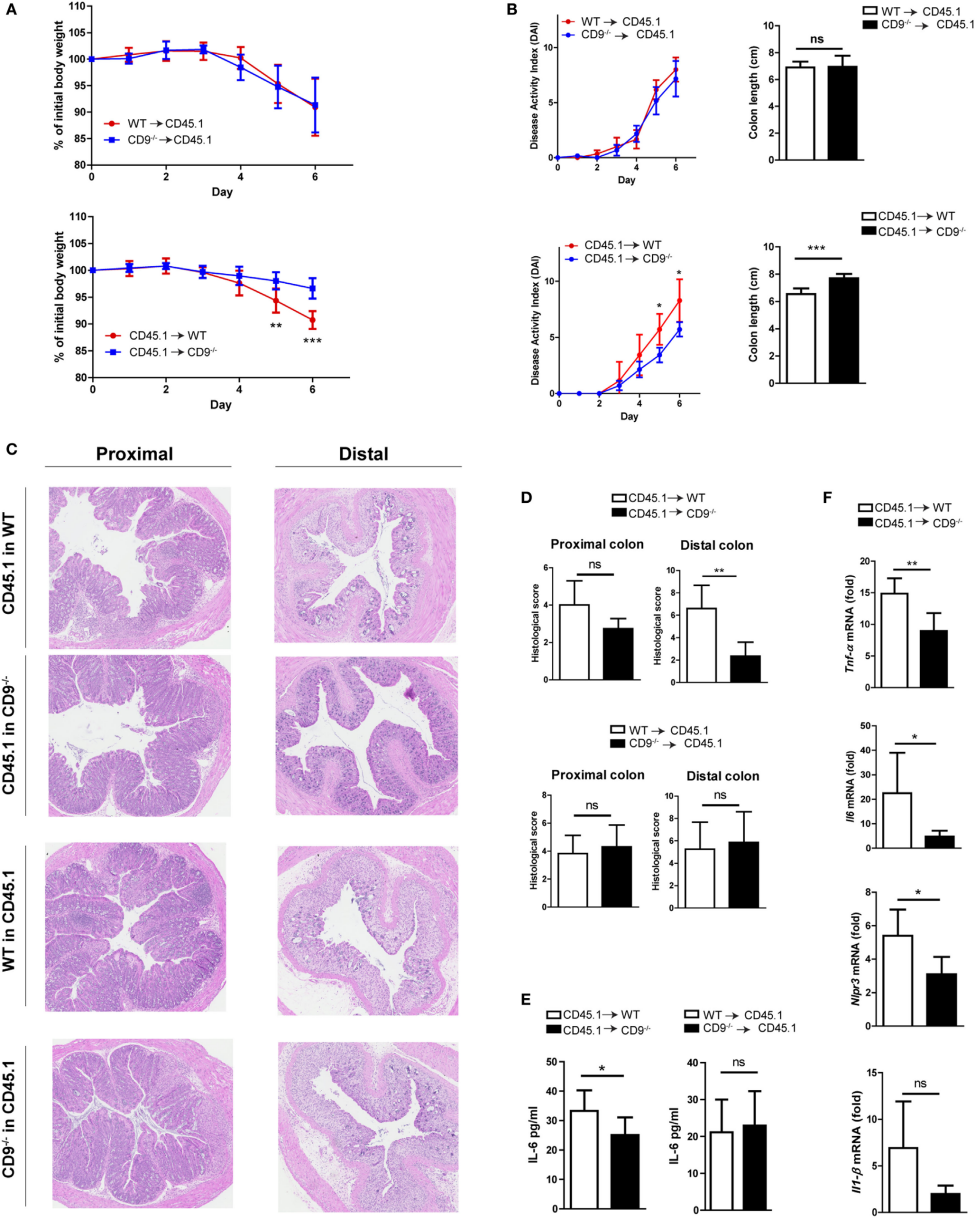
Lack of CD9 in the resident non-hematopoietic compartment confers the reduced susceptibility to dextran sodium sulfate (DSS)-mediated colitis. Lethally irradiated WT CD45.1 mice were rescued with WT or CD9^−/−^ CD45.2 bone marrow, whereas lethally irradiated WT and CD9^−/−^ CD45.2 mice were rescued with WT CD45.1 bone marrow. Three months post-transplantation mice were treated with 2% DSS. **(A)** Body weight evolution. **(B)** Disease activity index and colon shortening. **(C)** H&E stained proximal and distal colon sections. **(D)** Histological injury scores. **(E)** Serum IL-6 measured by ELISA. **(F)** qPCR analysis of proinflammatory cytokine expression in the colon of WT or CD9^−/−^ CD45.2 recipients. Experiments were perfomed twice, giving similar results. *n* = 6–7 per group. All between-group comparisons were analyzed by unpaired *t*-test; *P < 0.05; **P < 0.005; ***P < 0.001.

We next investigated the possible role of CD9 in myeloid cell populations or the resident non-hematopoietic cell compartment (mainly endothelial and epithelial cells). Two groups of chimeric mice were generated using the CD45.1 and CD45.2 haplotypes. Flow cytometry showed reconstitution levels of 95–99% (data not shown). Reconstitution experiments were carried out with WT CD45.1 mice and CD9^−/−^ or WT CD45.2 mice, with irradiation and transplantation in either direction. Protection against DSS-induced colitis was observed only when irradiated CD9^−/−^ mice were used as recipients of WT bone marrow (Figures 5A,B). Histology revealed typical DSS-induced changes in the distal and proximal colon of WT recipients and less pronounced alterations in CD9^−/−^ recipients reconstituted with CD45.1 WT bone marrow (Figures 5C,D). Only CD9^−/−^ recipients had lower DSS-induced levels of serum IL-6 measured by ELISA (Figure 5E), and colon samples from CD9^−/−^ recipients also had lower induced transcript expression of proinflammatory cytokines measured by qPCR (Figure 5F). These results underscore the conclusion that susceptibility to DSS-induced colitis is increased by CD9 expression in the non-hematopoietic compartment.

### Enhanced Colonocyte Proliferation after DSS-Induced Injury in CD9^−/−^ Mice

**Figure 6:**
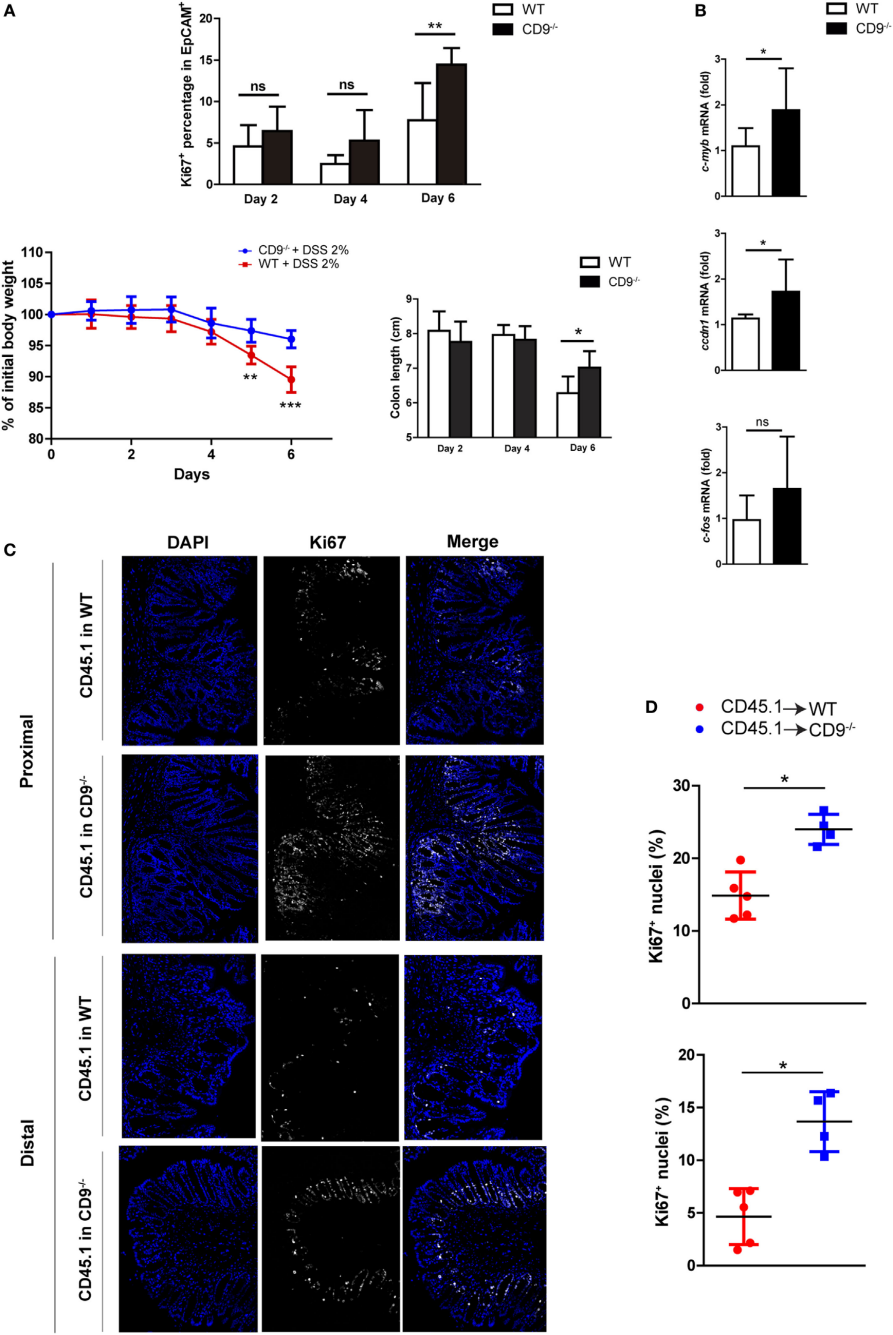
CD9 limits epithelial cell proliferation upon dextran sodium sulfate (DSS) challenge. **(A)** *Top*, FACS analysis of Ki67^+^ cells in the EpCAM^+^CD45^−^ gated population after days 2, 4, and 6 days 2% DSS exposure (upper panel). *Bottom*, body weight loss and colon shortening, *n* = 5–6 mice per group. **(B)** qPCR analysis of colonic mRNA expression of the cell-cycle genes *c-myb, ccdn1*, and *c-fos*. Data are pooled from two independent experiments, *n* = 4–6 mice per group. **(C)** Ki67 immunofluorescence staining on proximal and distal colon sections from untreated or 6-day DSS-treated chimeric mice. Representative magnification images are shown. **(D)** Quantification of Ki67^+^ cells in the epithelial layer of proximal (upper), and distal (lower) colons from 6-day DSS-fed chimeric mice. Each dot corresponds to the percentage of Ki67^+^ nuclei from all the epithelial nuclei of whole colon sections *n* = 4–5 mice per genotype. Between-group comparisons were analyzed by unpaired *t*-test for **(A,B)** and Mann–Whitney *U*-test for **(D)**; *P < 0.05; **P < 0.005; ***P < 0.001.

After DSS-induced epithelial cell damage, the colonic epithelium actively proliferates to restore intestinal barrier integrity. Flow cytometry analysis of the proliferation marker Ki67 in colonic EpCAM^+^ intestinal epithelial cells (IECs) from mice revealed that CD9 deficiency supports elevated colonic epithelial cell proliferation after DSS exposure (Figure 6A, top). However, no differences were detected in the proliferation of epithelial cells extracted from non-treated animals (Figure S2 in Supplementary Material). Remarkably, although the percentage of Ki67^+^ cells was slightly higher in CD9^−/−^ colon after 2 and 4 days of DSS exposure, the significant difference was observed at day 6. This is coincident with significant lower body weight loss and higher colon length in CD9^−/−^ mice (Figure 6A, bottom). Moreover, CD9^−/−^ colon showed higher mRNA expression of c-myc, c-fos, and cyclin D1 (Figure 6B). Analysis of the apoptosis marker caspase-3 was carried out by IHC in DSS-treated chimeric mice, with no differences between genotypes (Figure S3 in Supplementary Material). Proliferation in DSS-exposed colon of these animals was determined by counting immunostained Ki67^+^ cells in colon crypts on histological sections. The percentage of Ki67^+^ colonic cells was higher after DSS exposure in CD9^−/−^ recipients than in WT recipients (Figures 6C,D). Taken together, these results demonstrate that CD9 limits epithelial cell proliferation in response to injury.

## Discussion

Inflammatory bowel disease arises through close interaction between genetics, immunology, environment, and microbiome. The development and progression of this multifactorial disorder is affected by several factors, including diet, lifestyle, and behavior. Moreover, perturbations of the gut microbiota due to antibiotic medication may also play an important role in IBD. DSS-induced colitis has become a widely used model for studying IBD in the mouse (25, 26). DSS is a chemical colitogen toxic to gut epithelial cells, interfering with intestinal barrier function and stimulating local inflammation. This model is suitable for studying events triggered by temporary failure of mucosal homeostasis after epithelial cell shedding and loss of barrier integrity, and can also provide insight into the mechanisms that lead to MH after initial injury (27). Here, we report protection against DSS-induced colonic mucosal damage in CD9-deficient mice. These mice show lower DAI scores throughout treatment, larger colons, and have a less severe histological injury. The protection conferred by CD9 absence was confirmed by the increased survival of CD9^−/−^ mice upon administration of a lethal 4% DSS dose. Epithelial preservation *in vivo* was demonstrated by lower colonic transepithelial FITC-dextran leakage in CD9^−/−^ mice, and the importance of CD9 in the control of intestinal epithelial barrier function and integrity was further demonstrated by preserved expression of tight junction and other barrier-related genes in CD9^−/−^ mice.

CD9 is ubiquitously expressed, and we therefore performed chimeric reconstitution experiments to determine which cell compartment is responsible for mediating DSS-induced toxicity. Our data clearly demonstrated that protection in CD9^−/−^ animals was not dependent on the hematopoietic cell compartment. In CD9^−/−^ colon, crypt and villous distortion is minimal and surface epithelium is more preserved; this keeps luminal pathogens outside the lamina propria, and therefore proinflammatory cytokine and chemokine release is lower and there is less inflammatory cell recruitment. Specifically, the myeloid-derived cytokines involved in the inflammatory response in DSS acute colitis iNOs, TNF-α, IL-6, IL-12, and the inflammasome drivers NLRP3 and IL-1β were enhanced in WT mice versus CD9^−/−^ mice, but no differences were observed in either IFNγ, IL-17, or IL-22 cytokines. DSS colitis can be exacerbated by granulocyte recruitment (28–30). However, the reconstitution experiments ruled out a contribution to colon protection from CD9 deficiency in innate immune cells. The role of endothelial CD9 could not be completely discarded, and additional research with endothelium-specific deletion of CD9 would be required to resolve this issue. In addition, CD9 plays an important role in T cell activation (31–34). However, our data in the adoptive T cell transfer-mediated colitis model and *in vitro* T cell polyclonal experiments showed no significant differences in the differentiation and activation of CD9-deficient and WT Th1 and Th17 T cell subsets.

Flow cytometry and immunohistochemistry analysis revealed a higher percentage of Ki67 IECs in DSS-exposed CD9^−/−^ colon. In the distal colon, the percentage Ki67^+^ cells is lower than in the proximal colon. This likely reflects the more severe colitis in the middle and distal third of the colon in DSS-exposed mice, causing a predominantly distal injury characterized by epithelial ulceration and impaired regeneration (35). Notably, the difference in proliferation was observed after 6 days of DSS exposure, suggesting that it is related to mechanisms of post-injury epithelial recovery. CD9 absence thus does not increase IEC proliferation *per se* and only supports MH after injury. Indeed, hyperplasia and dysplasia were not observed in any CD9^−/−^ animals after cessation of DSS exposure.

Dextran sodium sulfate treatment leads to the exposure of the Toll-like receptors on the IEC basolateral surface. This triggers a proliferation that contributes to mucosal repair after injury, and DSS-induced colitis is exacerbated in mice with gene deletions affecting TLR signaling such as Tlr2^−/−^, Tlr4^−/−^, and Myd88^−/−^ (36–38). TLR signaling is linked to EGFR activation (39), which is required for intestinal homeostasis in the setting of acute mucosal damage (40, 41). CD9 could be playing several roles in these settings. EGFR signaling is increased in CD9-deficient cell lines (17, 42), and CD9 also negatively regulates ADAM17 (16) metalloproteinase activity, which is known to shed some EGFR ligands. CD9 deficiency will thus translate into increased EGFR phosphorylation and activation. Aside from a direct control of proliferation, CD9 might regulate epithelial restitution, its deficiency ultimately resulting in increased epithelial proliferation (43). In this context, CD9 absence might facilitate rapid resealing of the intestinal epithelial barrier and could promote IEC migration through impaired localization of talin-1 to focal adhesions (10) or increase CXCR4/CXCL12-mediated migration (44), a route that directly regulates epithelial cell migration, barrier maturation, and restitution (45). Compared with CD9-positive cells, CD9-negative or depleted epithelial and tumor cells have a much higher migratory capacity, thereby supporting epithelial restitution (42, 46).

Inflammatory bowel disease is effectively treated with anti-TNF-α monoclonal antibody (Infliximab), either as monotherapy or in combination with other immunomodulators, and current efforts are directed toward the crucial goal of achieving MH in order to accomplish long-term remission (4, 47). However, pharmacological anti-inflammatory agents such as glucocorticosteroids or 5-aminosalicylic acid do not heal the bowel mucosa (4), and the efficacy of growth factors such as GH and EGF has yet to be established (48). There is thus a clear need to identify new therapeutic targets for MH. Our results indicate that CD9 depletion enhances IEC proliferation, resulting in a high regenerative response and reduced susceptibility to DSS-induced colitis. Our findings thus reveal a critical role for the tetraspanin CD9 in colon inflammation and suggest a novel therapeutic opportunity. Growing recent evidence suggests that targeting tetraspanins by an array of tools including monoclonal antibodies, soluble large-loop proteins, and RNAi technology may be used to improve the course of IBDs.

## Ethics Statement

All animals were housed in pathogen-free conditions at the CNIC animal facility. Experimental procedures were approved by the local research ethics committee and conformed to EU Directive 2010/63EU and Recommendation 2007/526/EC, enforced in Spanish law under Real Decreto 53/2013.

## Author Contributions

MS designed research plan and performed all mice studies, analyzed and interpreted all data, and wrote the manuscript. DC helped to designed research plan and in mice experimentation, data interpretation, and writing of the manuscript. MR-H, DT, and OM-G performed experimental work. FS-M planned research, discussed results, and collaborated to write the manuscript.

## Conflict of Interest Statement

The authors declare that the research was conducted in the absence of any commercial or financial relationships that could be construed as a potential conflict of interest.
