# Supplementary material for: Tetraspanin CD9 Limits Mucosal Healing in Experimental Colitis
Source: Front Immunol. 2017 Dec 19;8:1854. doi: 10.3389/fimmu.2017.01854 (PMC5742144; doi:10.3389/fimmu.2017.01854)
Supplement: Supplementary file 1 [file Table_1.PDF]

Table S1. List of qPCR primers.

Gene	Forward primer	Reverse primer
Zo1	GAGCGGGCTACCTTACTGAAC	GTCATCTCTTTCCGAGGCATTAG
Tricellulin	TTCCGAAGCCTATCGTGATGC	GAACACAGCCTTATAGCGTTCT
Claudin-5	TATGAATCTGTGCTGGCGCT	GTGCTACCCGTGCCTTAACT
Claudin-7	CAGGCCACTCGAGCCTTAAT	GCAAGACCTGCCACAATGAAA
Claudin-8	GCAACCTACGCTCTTCAAATGG	TTCCCAGCGGTTCTCAAACAC
Claudin-10	AATCGTCGCCTTCGTAGTCTC	GTTGGCAAAATAAGTGGCTGTG
Muc1	GGCATTCGGGCTCCTTTCTT	TGGAGTGGTAGTCGATGCTAAG
Muc2	TGACTGCCGAGACTCCTACA	CCAGCTTGTGGGTGAGGTAG
Tff3c	GATTACGTTGGCCTGTCTCC	TGAAGCACCAGGGCACATTT
Il6	TAGTCCTTCCTACCCCAATTTCC	TTGGTCCTTAGCCACTCCTTC
Nlpr3	CGAGACCTCTGGGAAAAAGCT	GCATACCATAGAGGAATGTGATGTACA
Il1b	GCAACTGTTCCTGAACTCAACT	ATCTTTTGGGGTCCGTCAACT
Il12p35	TACTAGAGAGACTTCTTCCACAACAAGAG	TCTGGTACATCTTCAAGTCCTCATAGA
Il12p40	GGAAGCACGGCAGCAGAAT	AACTTGAGGGAGAAGTAGGAATGG
inos	CAGGAAGTAGGTGAGGGCT	AATCTTGGAGCGAGTTGTGG
Ifng	CGGCACAGTCATTGAAAGCC	TGCATCCTTTTTCGCCTTGC
Il17	TTTAACTCCCTTGGCGCAAAA	CTTTCCCTCCGCATTGACAC
Il22	ATGAGTTTTTCCCTTATGGGGAC	GCTGGAAGTTGGACACCTCAA
TNFa	CAGGCGGTGCCTATGTCTC	CGATCACCCCGAAGTTCAGTAG
Gapdh	AGCTTGTCATCAACGGGAAG	TTTGATGTTAGTGGGGTCTCG
c-myb	GAGCACCCAACTGTTCTCG	CACCAGGGGCCTGTTCTTAG
Ccdn1	GCGTACCCTGACACCAATCTC	CTCCTCTTCGCACTTCTGCTC
c-fos	CGGGTTTCAACGCCGACTA	TTGGCACTAGAGACGGACAGA
