# Supplementary figures and images for: Tetraspanin CD9 Limits Mucosal Healing in Experimental Colitis
Source: Front Immunol. 2017 Dec 19;8:1854. doi: 10.3389/fimmu.2017.01854 (PMC5742144; doi:10.3389/fimmu.2017.01854)

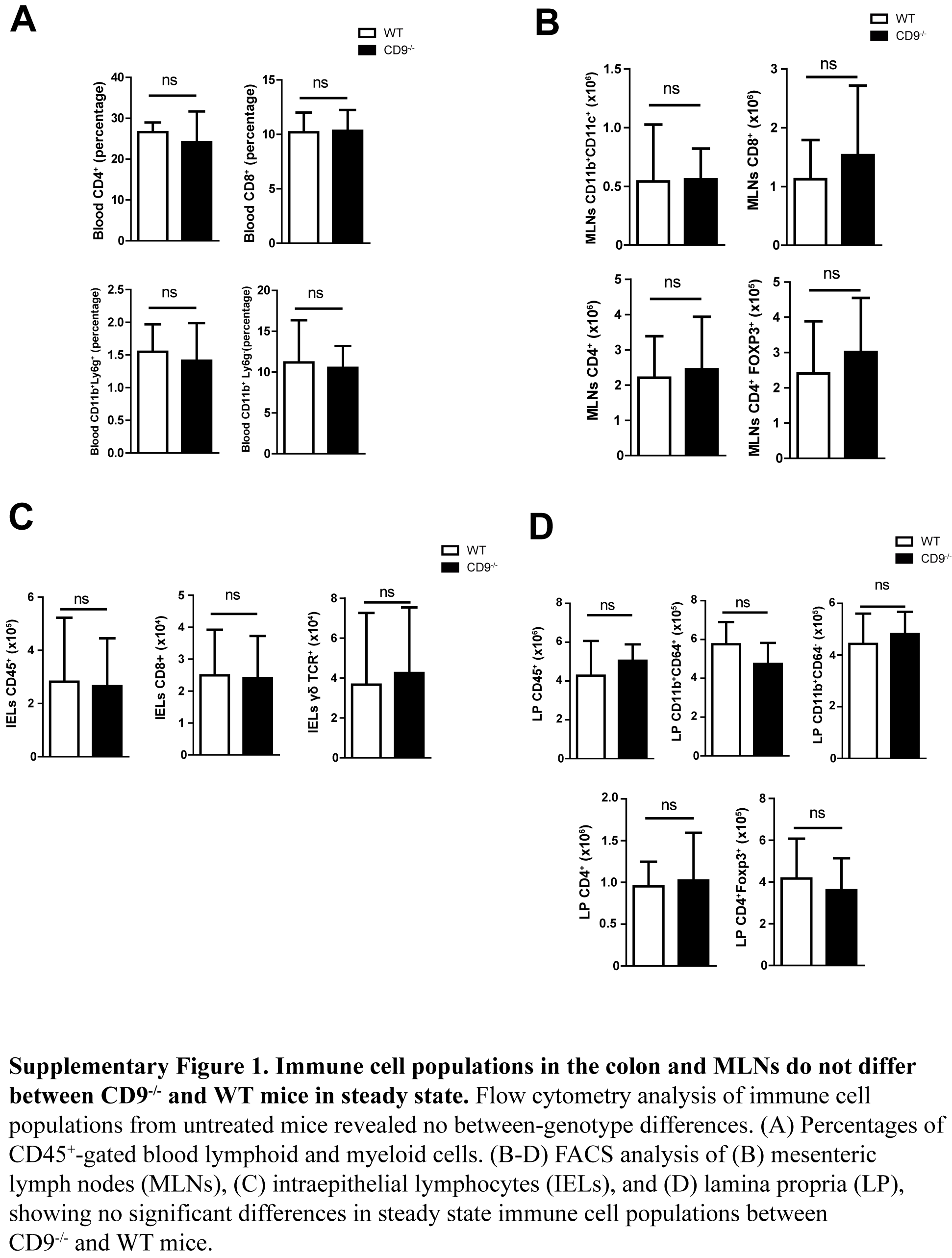

Supplement: Supplementary file 2 [file Image_1.tif]

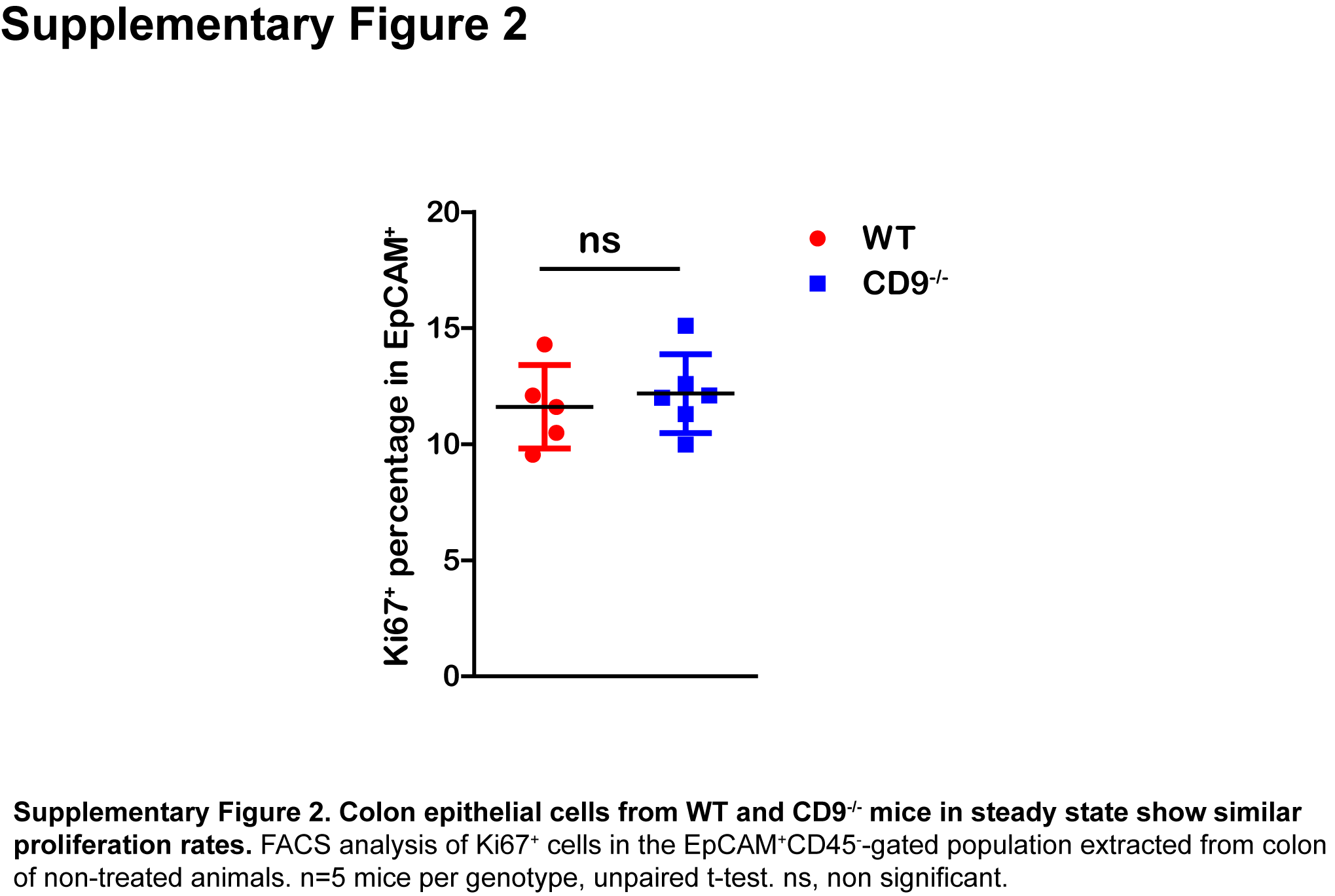

Supplement: Supplementary file 3 [file Image_2.tif]

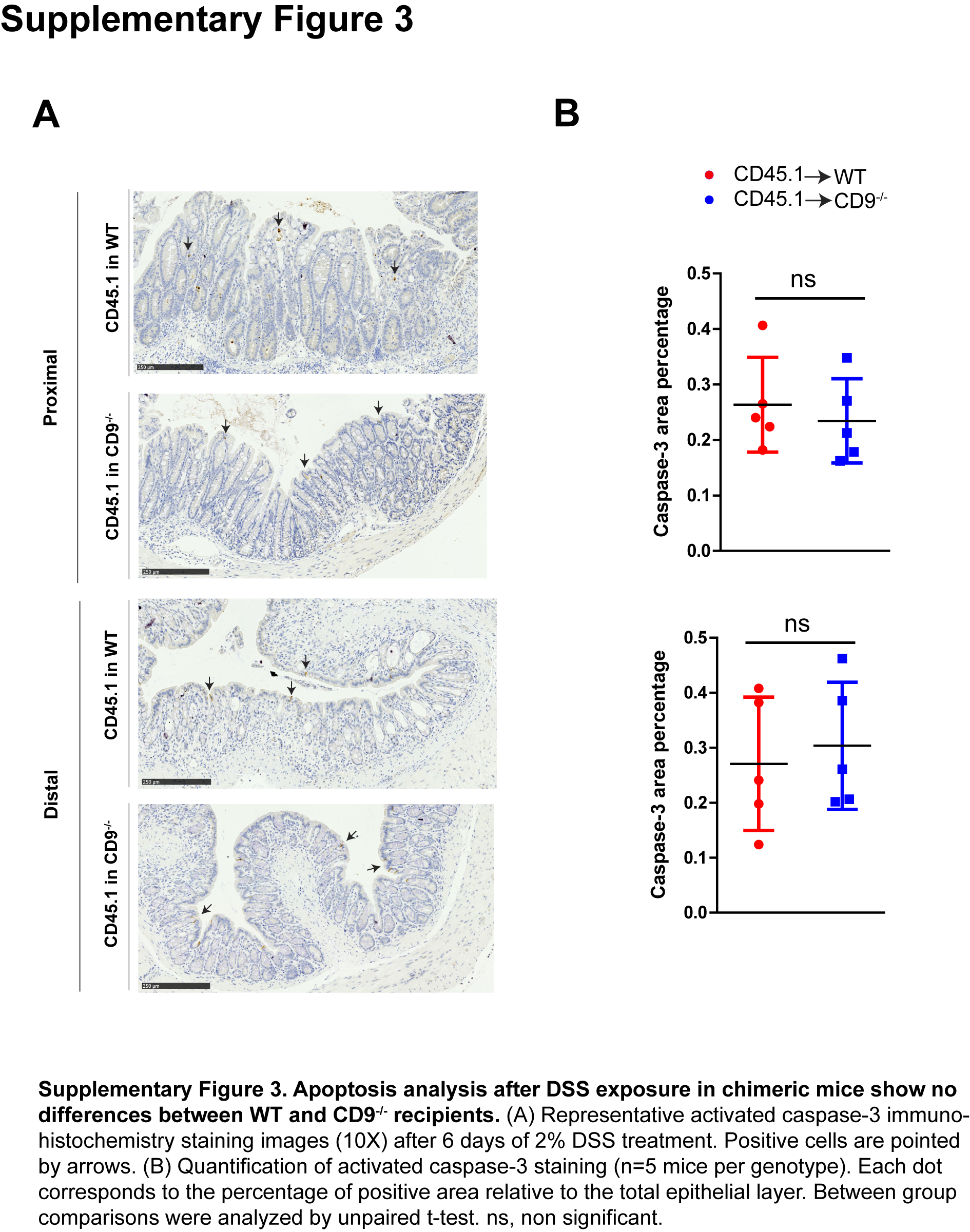

Supplement: Supplementary file 4 [file Image_3.tif]
